# A* Citrus bergamia* Extract Decreases Adipogenesis and Increases Lipolysis by Modulating PPAR Levels in Mesenchymal Stem Cells from Human Adipose Tissue

**DOI:** 10.1155/2016/4563815

**Published:** 2016-06-15

**Authors:** Debora Lo Furno, Adriana Carol Eleonora Graziano, Rosanna Avola, Rosario Giuffrida, Vincenzo Perciavalle, Francesco Bonina, Giuliana Mannino, Venera Cardile

**Affiliations:** ^1^Department of Biomedical and Biotechnological Sciences, Section of Physiology, University of Catania, Via S. Sofia 65, 95125 Catania, Italy; ^2^Department of Drug Sciences, University of Catania, Viale A. Doria 6, 95125 Catania, Italy

## Abstract

The aim of this research was to assess the impact of a well-characterized extract from* Citrus bergamia* juice on adipogenesis and/or lipolysis using mesenchymal stem cells from human adipose tissue as a cell model. To evaluate the effects on adipogenesis, some cell cultures were treated with adipogenic medium plus 10 or 100 *μ*g/mL of extract. To determine the properties on lipolysis, additional mesenchymal stem cells were cultured with adipogenic medium for 14 days and after this time added with* Citrus bergamia* for further 14 days. To verify adipogenic differentiation, oil red O staining at 7, 14, 21, and 28 days was performed. Moreover, the expression of peroxisome proliferator-activated receptor gamma (PPAR-*γ*), adipocytes fatty acid-binding protein (A-FABP), adipose triglyceride lipase (ATGL), hormone-sensitive lipase (HSL), monoglyceride lipase (MGL), 5′-adenosine monophosphate-activated protein kinase (AMPK)*α*1/2, and pAMPK*α*1/2 was evaluated by Western blot analysis and the release of glycerol by colorimetric assay.* Citrus bergamia* extract suppressed the accumulation of intracellular lipids in mesenchymal stem cells during adipogenic differentiation and promoted lipolysis by repressing the expression of adipogenic genes and activating lipolytic genes.* Citrus bergamia* extract could be a useful natural product for improving adipose mobilization in obesity-related disorders.

## 1. Introduction

Obesity, characterized by an excess accumulation of adipose tissue, has emerged as one of the leading public-health problems in the past decades. Excess adipose tissue increases the risk for a number of metabolic disorders such as atherosclerosis, hypertension, insulin resistance, and cancer [1]. Adipocyte-derived pro- and anti-inflammatory adipokines play key roles in the energy balance and homeostasis. Systemic inflammation, insulin resistance, and obesity-related metabolic disorders arise in response to increased production and secretion of proinflammatory adipokines [2]. Thus, obesity is a chronic low-grade inflammation, where a genetic predisposition, a modern sedentary life style, a reduced physical activity, and an unlimited offer of food have a major role [3, 4].

Actually, the most effective treatment for the weight loss is a drastic change in lifestyle, healthy diet, regular physical activity, and giving up smoking.

Several studies on cells, animals, and humans indicate dietary bioactive compounds increasing thermogenesis and energy expenditure, decreasing inflammation and oxidative stress, and supporting progress toward weight loss and/or metabolic disorders decrease [5, 6].

In recent years, the beneficial properties of the* Citrus bergamia* juice have had a growing interest and the potentiality of its health promoting substances has been the subject of research [7]. The juice of* Citrus bergamia* is a natural source of flavonoids, such as naringin, hesperidin, neohesperidin, and neoeriocitrin, playing a major role in human health protection [8, 9]. Some of our findings demonstrated a marked antioxidant/anti-inflammatory activity of an extract from* Citrus bergamia* on an* in vitro* model of inflamed skin keratinocytes [10]. Moreover, more recently, a clinical study reported that* Citrus bergamia* extract supplementation has beneficial effects on plasma lipid levels also in subjects with moderate hypercholesterolemia [11].

Obesity is related to inflammation and inflammatory cytokines are highly upregulated in adipose tissue of obese subjects [12–15]. Thus, we hypothesised that the extract from* Citrus bergamia* juice possessing anti-inflammatory propriety could affect adipose tissue dynamics and adipocyte differentiation. Therefore, the aim of this study was to evaluate the impact of a well-characterized extract from* Citrus bergamia* on adipocyte differentiation using mesenchymal stem cells from human adipose tissue as a cell model.

## 2. Materials and Methods

### 2.1. Samples Preparation and Determination of Flavonoids

*Citrus bergamia* juice was obtained from peeled-off Risso* et* Poteau fruits collected from plantations located in Reggio Calabria (Italy). The juice was then processed as described in Graziano et al. [10]. Pure standards (neoeriocitrin, naringin, and neohesperidin) were purchased from Extrasynthese (Genay Cedex, France) and used as a comparison with lab-made mixture. In any case, it is important to underline that none of the standards used alone gave results comparable to those of extract of* Citrus bergamia* containing a pool of flavonoid compounds.

### 2.2. Cultures of Mesenchymal Stem Cells

Adipose tissue was collected from 10 donors, 5 males and 5 females (from 22 to 30 years of age and mean body mass index of 29 ± 3) undergoing liposuction procedures. Lipoaspirates were obtained after the informed consent from the subjects, according to the laws of the Italian Government. The mesenchymal stem cells from lipoaspirates were obtained as reported in Lo Furno et al. [16].

### 2.3. Identification of Mesenchymal Stem Cells Markers

In order to detect the type of cells derived from lipoaspirates, flow cytometry was carried out as reported in Musumeci et al. [17].

### 2.4. Differentiation of Mesenchymal Stem Cells in Adipocytes

The ability of mesenchymal stem cells to differentiate towards the adipogenic line was examined in experiments where these were put in culture for 7, 14, 21, and 28 days. Some cells, as a control, were incubated in DMEM-1 g (10% FBS, 1% penicillin/streptomycin, 1% of MSC growth supplement). Additional cells were maintained with adipogenic medium (human MesenCult MSC Basal Medium plus Adipogenic Stimulatory Supplement; cat. #05401 and #05403, resp.; StemCell Technologies, Milan, Italy).

### 2.5. Treatments with* Citrus bergamia* Extract

To evaluate the effects on adipogenesis, some cell cultures with or without adipogenic medium were treated with 10 or 100 *μ*g/mL of characterized and lyophilized* Citrus bergamia* added to the medium every 2-3 days during medium changes for 28 days.

To determine the properties on lipolysis, additional cells were cultured with or without adipogenic medium for 14 days. After this time, they were added with 10 or 100 *μ*g/mL of* Citrus bergamia* for further 14 days.

### 2.6. Oil Red O Staining

To verify adipogenic differentiation of cell cultures, oil red O staining at 7, 14, 21, and 28 days was performed. Oil red O is a lysochrome diazo dye (fat-soluble) used for staining of neutral triglycerides and lipids on sections and cells. For this, cells in 6-well plates were gently rinsed with PBS and fixed in a 10% formalin-PBS solution for 1 hour. After removing this solution, 125 *μ*L of 0.3% oil red O-isopropyl alcohol was added to each well and kept at room temperature for 15 min to stain the cells. After 15 min, each well was washed three times, and the dye in the cells was eluted with 125 *μ*L of isopropyl alcohol. Then, 100 *μ*L of the eluate in each well was transferred to 96-well plates, and the optical density at *λ* = 550 nm was measured using a microplate reader.

### 2.7. Determination of Adipogenic and Lipolytic Markers

The expression of peroxisome proliferator-activated receptor gamma (PPAR-*γ*), adipocytes fatty acid-binding protein (A-FABP), adipose triglyceride lipase (ATGL), hormone-sensitive lipase (HSL), monoglyceride lipase (MGL), 5′-adenosine monophosphate-activated protein kinase (AMPK)*α*1/2, and pAMPK*α*1/2 was evaluated by Western blot analysis as described in Lo Furno et al. [16]. The antibodies used were anti-PPAR-*γ* (H-100: sc-7196, Santa Cruz Biotechnology, Santa Cruz, CA,) (dilution 1 : 300), anti-A-FABP (C-15: sc-18661) (dilution 1 : 300), anti-ATGL (H-144: sc-67355) (dilution 1 : 300), anti-HSL (H-300: sc-25843) (dilution 1 : 200), anti-MGL (H-300: sc-134789) (dilution 1 : 200), anti-AMPK*α*1/2 (H300: sc-25792) (dilution 1 : 200), anti-p-AMPK*α*1/2 (Thr 172: sc-33524) (dilution 1 : 300), and anti-*α*-tubulin (T9026; Sigma–Aldrich) (1 : 5000 dilution). The signal intensity of primary antibody binding was analyzed quantitatively with ImageJ software and was normalized to a loading control *α*-tubulin. Values were expressed as arbitrary densitometric units (ADU) corresponding (proportional) to signal intensity.

### 2.8. Glycerol Assay

Glycerol determination was performed by colorimetric assay kit from Cayman (item number 10010755) that measures glycerol by a coupled enzymatic reaction system. Glycerol is phosphorylated by glycerol kinase to produce glycerol-3-phosphate and adenosine-5′-diphosphate. The glycerol-3-phosphate is oxidized by glycerol phosphate oxidase producing dihydroxyacetone phosphate and hydrogen peroxide (H_2_O_2_). Peroxidase catalyses the redox-coupled reaction of H_2_O_2_ with 4-aminoantipyrine and N-ethyl-N-(3-sulfopropyl)-m-anisidine, producing a brilliant purple product with an absorbance maximum at *λ* = 540 nm. The results were expressed as *μ*g/L glycerol (dynamic range of the kit was 0–20 *μ*g/L glycerol).

### 2.9. Cell Viability Assay

The effect on cell viability of* Citrus bergamia *was evaluated with a test based on the cleavage of 3-(4,5-dimethyl-2 thiazolyl)-2,5-diphenyl-2H-tetrazolium bromide (MTT) by mitochondrial dehydrogenases of metabolically active cells [18]. The optical density at *λ* = 550 nm was measured using a microplate reader.

### 2.10. Statistical Analysis

All data were presented as mean ± SD of at least three separate experiments. The statistical analysis was performed by using two-way ANOVA followed by Dunnett's* post hoc* test for multiple comparisons with control. All statistical analyses were performed using the statistical software package SYSTAT, version 11 (Systat Inc., Evanston IL, USA). The unpaired Student's *t*-test was used to compare two different groups. A *p* value <0.05 was considered to be statistically significant.

## 3. Results

### 3.1. Determination of Flavonoids

The flavonoids profile of* Citrus bergamia* extract enriched in polyphenols was reported in Graziano et al. [10]. Briefly, the main flavonoids identified by HPLC and HPLC-MS were apigenin 6,8-di* C*-glucoside, diosmetin 6,8-di* C*-glucoside, neoeriocitrin, naringin, and neohesperidin. The total amount of flavonoids in* Citrus bergamia* solid extract was 25–27% w/w. The content of flavonoids, according to HPLC analysis, was as follows: neoeriocitrin (5.2 ± 0.3%), naringin (8.9 ± 0.2%), neohesperidin (13.1 ± 0.3%), and other flavonoids (5 ± 0.8%).

### 3.2. Identification of Mesenchymal Stem Cells Markers

After the first passage, flow cytometry analysis demonstrated that mesenchymal stem cells did not present labeling for hematopoietic line markers (CD45, CD14, and CD34) and were positive for CD44, CD90, and CD105 (Table 1).

### 3.3. Effect of* Citrus bergamia* on Cell Viability

**Figure 1:**
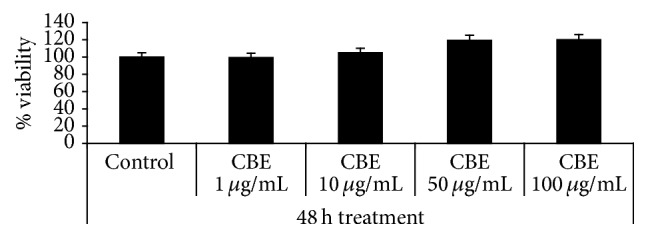
Effects of* Citrus bergamia* extract (CBE) on mesenchymal stem cells viability, determined using the 3-(4,5-dimethyl-2 thiazolyl)-2,5-diphenyl-2H-tetrazolium bromide (MTT) assay, after 48 hours of treatment with 1, 10, 50, or 100 *μ*g/mL of CBE. The values of optical density measured at *λ* = 550 nm are reported as percentage with respect to the optical density registered for untreated control (Control), the latter considered as 100% of cell viability. The values are mean ± SD of three experiments performed in triplicate.

The results of the MTT assay (Figure 1) showed that* Citrus bergamia* has no effect on cell viability, because treatment with 1, 10, 50, and 100 *μ*g/mL for 48 hours did not reduce the ability of stem cell cultures to metabolise tetrazolium salts at any concentration.

### 3.4. Effect of* Citrus bergamia* on Adipogenesis or Lipolysis

**Figure 2:**
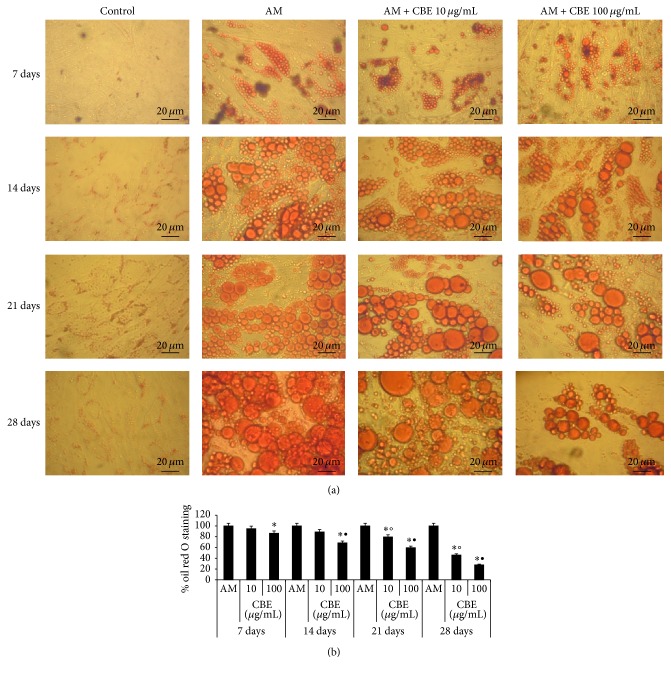
Effect of 10 and 100 *μ*g/mL* Citrus bergamia* extract (CBE) on the lipid content of mesenchymal stem cells stained with oil red O during adipogenic differentiation. Cells were examined at 7, 14, 21, and 28 days from the start of differentiation. (a) A representative photograph from three independent experiments of untreated control (Control), adipogenic medium-treated cells (AM), and AM plus 10 or 100 *μ*g/mL CBE was shown. (b) The levels of oil red O incorporation were quantified by measuring the absorbance of isopropyl alcohol extract at *λ* = 550 nm. Data were shown relative to AM and expressed as mean ± SD from three independent experiments. ^*∗*^ *p* < 0.01 versus AM (100%) at same day; °*p* < 0.05 versus CBE 10 *μ*g/mL at 7 days; ^•^ *p* < 0.01 versus CBE 100 *μ*g/mL at 7 days.

Adipocytes were verified by the presence of intracellular vesicles containing lipid by means of staining with oil red O. In the control cells there was no staining while in the mesenchymal stem cells treated with adipogenic medium it was possible to appreciate the formation of large vacuoles containing lipids in which it was evident to be a red-orange staining (Figure 2(a)), which increased in size in a time-dependent manner. The number of these cells increased continuously up to the 28th day of culture (Figure 2(a)). More than 60% of human mesenchymal stem cells were differentiated into adipocytes within the four-week induction period. Differently, the cell cultures with adipogenic medium treated with 10 or 100 *μ*g/mL of* Citrus bergamia* showed a decrease in the number and size of intracellular lipid vacuoles in a concentration-dependent manner (Figure 2(a)).

**Figure 3:**
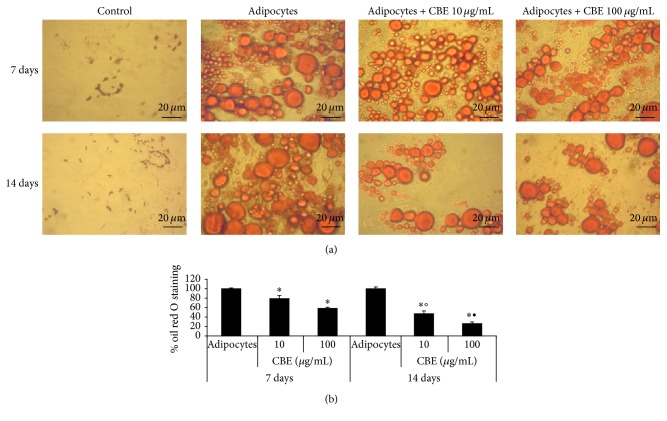
Effect of 10 and 100 *μ*g/mL* Citrus bergamia* extract (CBE) on the lipid content of 14-day differentiated adipocytes stained with oil red O. (a) A representative photograph from three independent experiments of untreated mesenchymal stem cells (Control), 14-day differentiated cells (adipocytes), adipocytes with 10 or 100 *μ*g/mL CBE at 7 and 14 days was shown. (b) The levels of oil red O incorporation was quantified by measuring the absorbance of isopropyl alcohol extract at *λ* = 550 nm. Data were shown relative to 14-day differentiated adipocytes and expressed as mean ± SD from three independent experiments. ^*∗*^ *p* < 0.01 versus the 14-day differentiated adipocytes (100%) at same day; °*p* < 0.01 versus CBE 10 *μ*g/mL at 7 days; ^•^ *p* < 0.01 versus CBE 100 *μ*g/mL at 7 days.

Compared to the differentiated adipocytes, mesenchymal stem cells cultured with adipogenic medium for 14 days and after this time added with 10 or 100 *μ*g/mL of* Citrus bergamia* for further 14 days displayed a lower number of smaller size vacuoles (Figure 3(a)). Oil red O lipid quantification confirmed that* Citrus bergamia* decreased lipid accumulation in a time- and concentration-dependent manner (Figures 2(b) and 3(b)).

### 3.5. Determination of Adipogenesis Markers

**Figure 4:**
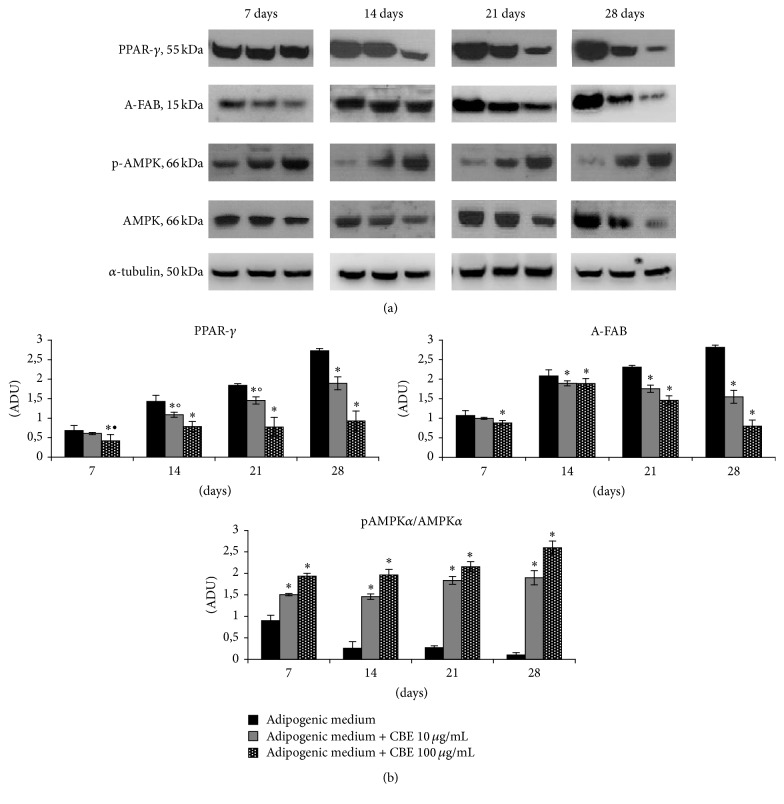
Inhibitory effects of 10 or 100 *μ*g/mL* Citrus bergamia *extract (CBE) on adipocyte differentiation in mesenchymal stem cells induced to differentiate into adipocytes by using adipogenic medium in presence or absence of CBE. At 7, 14, 21, and 28 days, differentiated mesenchymal stem cells adipocytes were examined for PPAR-*γ*, A-FAB, AMPK*α*1/2, and p-AMPK*α*1/2 by Western blot. The levels of proteins were expressed as arbitrary densitometric units (ADU) and the ratio p-AMPK*α*1/2/AMPK*α*1/2. Data are shown relative to adipogenic-treated cells and expressed as mean ± SD from three independent experiments. ^*∗*^ *p* < 0.01 versus the mesenchymal stem cells treated with adipogenic medium at same day; °*p* < 0.01 versus CBE 10 *μ*g/mL at 28 days; ^•^ *p* < 0.01 versus CBE 100 *μ*g/mL at 28 days.

To elucidate the mechanism of the differentiation inhibitory effect of* Citrus bergamia*, the protein expression levels of PPAR-*γ*, A-FABP, and AMPK*α*1/2 were evaluated by Western blot analysis at 7, 14, 21, and 28 days in differently treated cells. PPAR-*γ* was weakly detected in undifferentiated cells, and its expression level increased in time-dependent manner during adipocyte differentiation, as compared with that in the undifferentiated cells (Figure 4). In contrast, when the cells were caused to differentiate into adipocytes in medium containing* Citrus bergamia *at 10 or 100 *μ*g/mL, the expression of PPAR-*γ* was reduced. At 100 *μ*g/mL, the levels for PPAR-*γ* were decreased to approximately 39, 45, 58, and 66% at 7, 14, 21, and 28 days, respectively, compared to the differentiated cells (Figure 4).

A-FABP is a cytoplasmatic protein highly expressed in adipocytes. Our results showed that, compared to mesenchymal stem cells differentiated with adipogenic medium for 28 days, incubation with* Citrus bergamia* decreased the total A-FABP content in a time- and concentration-dependent manner (Figure 4).

To elucidate the molecular mechanism of the suppressive effect of* Citrus bergamia* extract on adipogenesis, we investigated the possibility of* Citrus bergamia* acting as an activator of AMPK*α*1/2. It is known that the activation of AMPK suppresses adipogenesis [19]. AMPK*α*1/2 was expressed constitutively even in the absence of* Citrus bergamia*. Phosphorylation of AMPK*α*1/2 was enhanced when the cells were cultured in DMEM containing 10 or 100 *μ*g/mL* Citrus bergamia*, although the AMPK*α*1/2 level in any sample was not altered by this treatment (Figure 4). Furthermore, the efficiency of phosphorylation of AMPK at 7 days was similar than that at 28 days, revealing that* Citrus bergamia* rapidly phosphorylated AMPK*α*1/2 in these cells.

### 3.6. Determination of Lipolysis Markers

**Figure 5:**
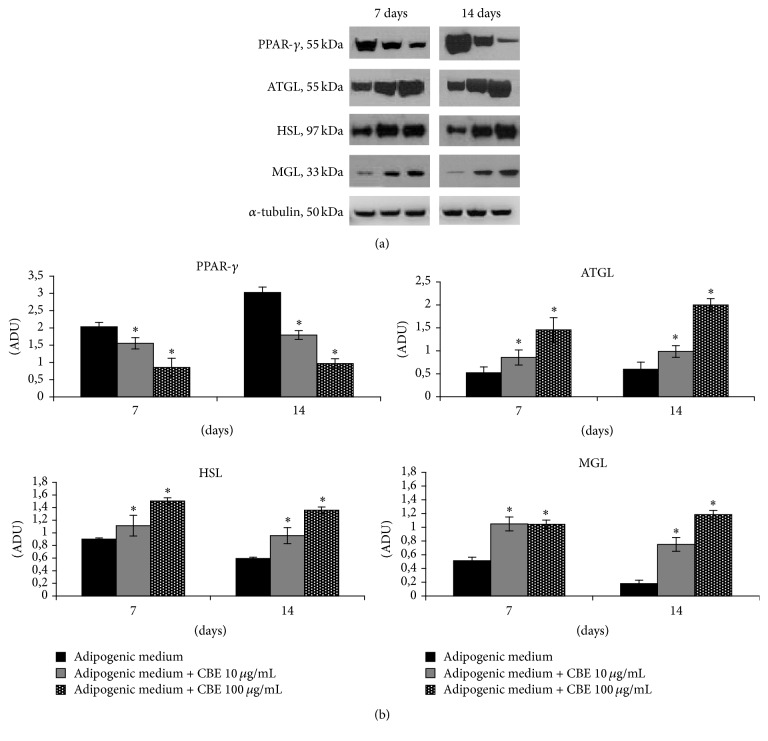
Effects of* Citrus bergamia* extraction on PPAR-*γ*, ATGL, HSL, and MGL: 14-days differentiated adipocytes were treated with 10 or 100 *μ*g/mL for 7 or 14 days. The levels of proteins are expressed as arbitrary densitometric unit (ADU). Data were shown relative to 14-day differentiated adipocytes (adipocytes) and expressed as mean ± SD from three independent experiments. ^*∗*^ *p* < 0.01 versus the 14-day differentiated adipocytes (100%).

Differentiated adipocytes from mesenchymal stem cells cultured with adipogenic medium for 14 days and after this time treated with 10 or 100 *μ*g/mL of* Citrus bergamia* for further 14 days displayed a concentration- and time-dependent decrease of PPAR-*γ* (Figure 5).

Moreover, we examined the expression of genes involved in the lipolysis such as ATGL, HSL, and MGL and measured glycerol release from the cells treated or not with* Citrus bergamia* extract.

The production of ATGL, HSL, and MGL was upregulated by* Citrus bergamia* extract in a time- and concentration-dependent manner, as compared with that of the 14-day differentiated adipocytes (Figure 5).

**Figure 6:**
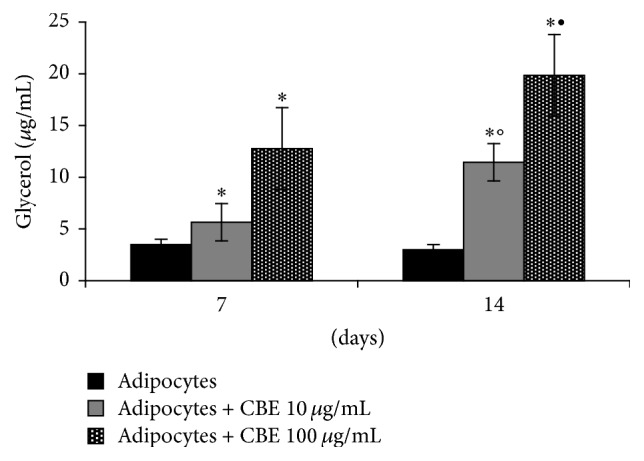
Stimulation of lipolysis by 10 or 100 *μ*g/mL* Citrus bergamia* extract (CBE) in 14-day differentiated adipocytes, assessed by the amount of glycerol released into the media. Data were expressed (*μ*g/mL) as mean ± SD from a representative triplicate experiment. ^*∗*^ *p* < 0.01 versus the 14-day differentiated adipocytes; °*p* < 0.05 versus CBE 10 *μ*g/mL at 7 days; ^•^ *p* < 0.01 versus CBE 100 *μ*g/mL at 7 days.

Furthermore, the treatment of 14-day differentiated adipocytes with 10 or 100 *μ*g/mL of* Citrus bergamia* extract significantly increased the content of free glycerol to 8 ± 2 and 19 ± 4 *μ*g/mL, respectively, as compared to control cells at 3 ± 0.5 *μ*g/mL (Figure 6).

## 4. Discussion

In this study, we reveal that a* Citrus bergamia* extract suppressed the accumulation of intracellular lipids in mesenchymal stem cells during adipogenic differentiation and promoted lipolysis by repressing the expression of adipogenic genes and activating lipolytic genes. The* Citrus bergamia*-mediated suppression of adipogenesis occurred through decrease of PPAR-*γ* and A-FABP and activation of AMPK. The* Citrus bergamia*-mediated activation of lipolysis occurred through increase of ATGL, HSL, and MGL expression and glycerol release.

It is well known that obesity occurs because of an imbalance between energy intake and expenditure, leading to increased numbers of adipocytes (hyperplasia) and increased adipocytes size (hypertrophy) [2]. On the contrary, lipolysis is one of the most important mechanisms to reduce adipose mass, leading to the breakdown of triacylglycerol stored in adipocytes and release of free fatty acids and glycerol [3]. Accordingly, the inhibition of the differentiation and proliferation of adipocytes and/or the promotion of fat mobilization in adipocytes could be used as a strategy for the treatment and prevention of obesity.

Several natural products have been reported to be involved in the suppression of adipogenesis [19]. Among these, flavonoids are naturally occurring polyphenolic compounds present in a variety of fruits, vegetables, and seeds [19]. These products possess many biological, pharmacological, antioxidant, and antimicrobial activities [20–22].

Citrus is one of the most important fruit crops worldwide and is rich in nutrients and bioactive compounds. Citrus fruits contain not only basic nutrient compounds such as vitamins, minerals, pectins, and dietary fibers, but also ample bioactive compounds including flavonoids, carotenoids, limonoids, and coumarins. In recent years, the study on the use of citrus fruits in the prevention and treatment of obesity and its related metabolic diseases has attracted increasing attention [23–27].

*Citrus bergamia*, commonly named bergamot, is a fruit mainly used for its essential oil extracted from the peel in the pharmaceutical industry, in the cosmetic industry, and in food industries as aroma. On the contrary, the juice because of its bitter taste has not found a real use in the industry and it is considered a waste of the essential oil production. However, the juice is a natural source of flavonoids, especially in terms of naringin, hesperidin, neohesperidin, and neoeriocitrin [18]. In this research, we demonstrated that* Citrus bergamia* extract affects adipogenesis and lipolysis giving a new useful suggestion for the industry on use of bergamot juice, which otherwise would be an unusable waste product.

Adipocyte differentiation is regulated by a complex mechanism including transcriptional regulation for coordinate changes in the expression of adipocyte-specific genes [28]. PPAR-*γ* is a key nuclear receptor transcription factor in adipogenesis and lipogenesis [29–33]. It regulates the expressions of the genes related to fatty acid oxidation, synthesis, and adipogenesis. Our results indicated that* Citrus bergamia* extract decreased the expression of PPAR-*γ* suppressing the differentiation of the preadipocytes into adipocytes.

It is known that activated AMPK attenuates adipogenesis including the synthesis of glycerol lipids and augments fatty acid oxidation [34, 35]. It was reported also that AMPK can be activated by flavonoids such as quercetin and epigallocatechin gallate [32], both of which suppress adipogenesis. Regarding the molecular mechanism, Thr172 residue of AMPK *α*-subunits is phosphorylated by liver kinase B1 or by calcium/calmodulin-dependent protein kinase kinase-*β* [36, 37].

Our data showed that* Citrus bergamia* extract enhanced the phosphorylation of AMPK in mesenchymal stem cells, indicating that it acts as an activator of AMPK. Probably,* Citrus bergamia* extract modulates the activity of calcium/calmodulin-dependent protein kinase kinase-*β*, triggers the AMPK cascade in adipocytes, and attenuates the progression of adipogenesis.

In summary, we presently show that* Citrus bergamia* suppressed adipogenesis through downregulation of PPAR-*γ* function by activating AMPK in adipocytes differentiated from mesenchymal stem cells. Thus,* Citrus bergamia* extract has the potential for use as an antiadipogenic agent to lower the content of body fat and prevent a gain in body weight. It is also necessary to underline that the 3T3-L1 cells have been used as a model in the majority of the* in vitro* studies published until now [38–40]. In the present research, we used mesenchymal stem cells from human adipose tissue confirming them as a good model to study the effects of polyphenols or other natural compounds on obesity.

## 5. Conclusions

In conclusion, the results of this study showed that* Citrus bergamia* extract, containing a good pool of compounds such as neoeriocitrin, naringin, neohesperidin, and other flavonoids, effectively reduces the level of intracellular neutral lipid by promoting triglycerides metabolism in order to diminish fat stores in adipocytes, thereby combating obesity. Additionally,* Citrus bergamia* extract displayed a significant inhibitory effect on adipogenesis, reducing a capacity to produce mature adipocytes from mesenchymal stem cells and regulating adipose tissue mass.

Therefore,* Citrus bergamia* extract could be a useful natural product for improving adipose mobilization in obesity-related disorders.

## Figures and Tables

**Table 1:** Mesenchymal stem cells markers identified by flow cytometric analysis.

Cell surface (cluster of differentiation) marker	% positive cells
CD44	95.5 ± 0.3
CD90	88.6 ± 0.5
CD105	80.2 ± 1.6
CD14	8.3 ± 6.2
CD34	5.5 ± 6.3
CD45	9.7 ± 6.2
